# Examining health promotion interventions for patients with chronic conditions using a novel patient-centered complexity model: protocol for a systematic review and meta-analysis

**DOI:** 10.1186/2046-4053-2-29

**Published:** 2013-05-11

**Authors:** Amy E Bodde, Nathan D Shippee, Carl R May, Frances S Mair, Patricia J Erwin, M Hassan Murad, Victor M Montori

**Affiliations:** 1 Knowledge and Evaluation Research Unit, Mayo Clinic, 200 First Street SW, Rochester, MN, 55905, USA; 2 Division of Health Care Policy and Research, Mayo Clinic, Rochester, MN, USA; 3 Division of Health Policy and Management, Mayo Building A302, MMC 197 420 Delaware Street SE, Minneapolis, MN, 55455, USA; 4 Health Sciences, University of Southampton, University Road, Southampton, SO17 1BJ, UK; 5 General Practice and Primary Care, Institute of Health and Wellbeing, University of Glasgow, 1055 Great Western Road, Glasgow, G12 0XH, UK; 6 Division of Preventive, Occupational and Aerospace Medicine, Mayo Clinic, Rochester, MN, USA; 7 Division of Endocrinology, Diabetes, Metabolism, Nutrition, Mayo Clinic, Rochester, MN, USA

**Keywords:** Health behavior, Comorbidities, Multimorbidity, Chronic conditions, Patient complexity, Cumulative complexity model, Physical activity, Diet

## Abstract

**Background:**

Successful chronic care self-management requires adherence to healthy lifestyle behaviors, but many healthcare-based health promotion interventions have resulted in small and unsustainable changes in patient behavior. Patients with chronic conditions may already be overwhelmed by burdensome illnesses and treatments, and not have the capacity to respond well to the additional work required of behavior modifications. To explore this phenomenon, we will apply the cumulative complexity model (CCM), a patient-centered model of patient complexity, to a systematic review and meta-analysis of healthcare-based health behavior interventions.

**Methods/Design:**

This systematic review will include randomized trials published between 2002 and 2012 that compared healthcare-based interventions aimed at improving healthy diet and physical activity in community dwelling adult patients with chronic conditions. After extracting study and risk of bias features from each trial, we will classify the interventions according to the conceptual model. We will then use meta-analysis and subgroup analysis to test hypotheses based on the conceptual model.

**Discussion:**

Healthcare providers need evidence of successful health promoting interventions for patients with chronic conditions who display common behavioral risk factors. To better understand how patients respond to interventions, we will apply the CCM*,* which accounts for both the capacity of patients with chronic conditions and their treatment-related workload, and posits that a balance between capacity and workload predicts successful enactment of self-care. Analysis will also include whether patients with multiple chronic conditions respond differently to interventions compared to those with single chronic conditions. The results of this review will provide insights as to how patients with chronic conditions respond to health-promoting interventions.

**Review registration:**

PROSPERO registration number: CRD42012003428

## Background

Chronic health conditions afflict nearly 50% of the USA population and cause 70% of deaths in the USA each year [1,2]. The care of individuals with chronic conditions currently accounts for 78% of all healthcare spending in the USA, which is only expected to increase [3-5]. Multimorbidity, defined as two or more chronic conditions in an individual, adds even more complexity and burden to patients and the healthcare system. Approximately 21% to 23% of adults are reported to be multimorbid, and the majority of people aged 65 and over have multimorbidity [6,7]. Individuals with multimorbidity face functional impairment earlier than those without [8], and have health consequences beyond the additive effect of each condition [5,9].

Patients with chronic conditions face many treatment demands, including managing numerous appointments, adhering to medications and self-monitoring their conditions [10-12], along with needing to practice important health behaviors, such as maintaining a healthy diet and physical activity. These demands may be further compounded by financial concerns, complex medical regimens, low health literacy, poor self-efficacy and fears about treatments [13-15]. Moreover, care is complicated by mental health disorders, especially depression, the prevalence of which increases as the number of chronic conditions increases [7,16]. The prevalence of multimorbidity is escalating and these added burdens substantially affect chronic disease self-management [17,18].

Since patients with chronic conditions are high utilizers of healthcare services [19], healthcare providers are poised to influence their patients’ health and self-management behaviors. However, previous reviews have found that even effective healthcare-delivered interventions are often very complex and effect sizes are small [20]. Further, since behavior change interventions often simultaneously employ numerous techniques to promote health behaviors, it is difficult to discern which of the intervention components are the most effective.

### Previous reviews and knowledge gaps

Past reviews of healthcare-delivered behavior change interventions have evaluated the results and quality of interventions, but provided little insight into how they may differentially affect patients with chronic conditions for whom behavior change may be especially demanding. Reviews of interventions to improve health behaviors among patients (regardless of chronic condition status) have found inadequate evidence to recommend interventions for diet and physical activity [21-24]. Many reviews of behavior change interventions have been behavior-specific or disease-specific, instead of assessing generic approaches which may be needed for the increasing number of patients presenting with multimorbidity and concomitant multiple behavioral risk factors [20,25]. Other reviews of interventions for patients have focused only on counseling and communication techniques, and excluded other possible practice innovations [26]. Reviews of comprehensive care and chronic disease self-management programs, in which behavior interventions are often nestled, have found some positive effects on behaviors, such as physical activity; however, many of these reviewed trials have not adequately measured or reported behavior outcomes [27-29]. Often, behavioral intervention trials indicate that the most time- and contact-intensive interventions result in better outcomes for patients; however, these may be less acceptable because they are burdensome to the patient and provider, and are inconvenient for long-term maintenance [20].

Here, research on the experiences of patients with chronic conditions is informative. Since patients with chronic conditions are already undertaking considerable work to understand their illness and treatments, engage with others to organize care, and adhere to and monitor treatments [11,12], they may have diminished capacity to enact health behavior changes as well. The added burdens of having multimorbidity (polypharmacy, monitoring for interactions and managing advice from multiple providers) may further impair adherence to treatments and behaviors, resulting in poorer health and reduced quality of life [11]. The status quo design of health behavior interventions may not take into account the capacity required of the patient to perform the desired behaviors nor the unsustainable workload that the intervention places upon the patient.

There is mixed evidence for health behavior interventions for patients with chronic conditions and little evidence on the efficacy of behavior change interventions for patients with multimorbidity [29]. Thus, interventions to improve health behavior adherence among patients with chronic conditions, and especially those which address the challenges of multimorbidity, are essential.

**Figure 1:**
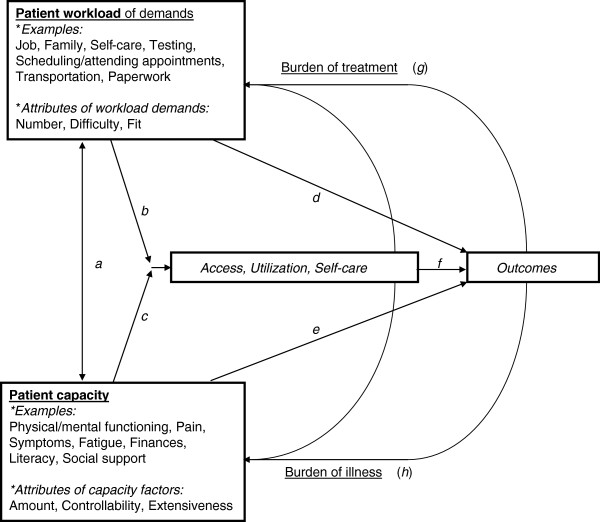
The cumulative complexity model (CCM).

Our intent is to conduct a systematic review and meta-analysis of healthcare interventions aimed at improving physical activity and diet among patients with chronic conditions, with a special focus on patients with multimorbidity. The innovation of this review is two-fold. First, we will assess how interventions impact patients with single conditions versus multimorbidity. Second, we will apply the cumulative complexity model (CCM) [30], a patient-centered conceptual model of patient complexity, to analyze intervention components in terms of how they: a. reduce patients’ workloads of treatment and self-care demands, such as with reminders or simplification of regimens; and/or b. improve their capacity to manage demands through education, skill-building, long-term reductions in illness burden, or other approaches. These design considerations are especially important considering that intervention components themselves may incur at least short-term demands on patients’ time and effort (Figure 1).

We hypothesize that multimorbidity will be largely unaddressed in behavior change interventions, and that in studies which do account for multimorbidity, patients with multimorbidity will receive less benefit from interventions than those with single conditions. We also hypothesize that interventions with components which both reduce patients’ treatment-related workloads and bolster their capacity are more effective than interventions which do only one of the two, or than interventions which only add demands upon patients by requiring intensive behavior change with little or no support. Unlike previous systematic reviews, here we will overlay a patient-centered conceptual framework to examine the results of the reviewed studies which will add insight as to how healthcare interventions are experienced by patients with chronic conditions.

### Aims

Four primary research questions will be addressed in this review:

1. To what extent do healthcare interventions improve adherence to selected health behaviors in patients with chronic conditions?

2. Are intervention designs or results different for patients with single conditions versus multimorbidity?

3. How do workload-focused interventions compare to capacity-focused interventions?

4. Can the impact of these healthcare interventions be explained by the extent to which the intervention favorably affects the workload-to-capacity ratio of the patient?

## Methods/Design

The review team is multi-disciplinary and includes content experts, a reference librarian, clinician researchers, and systematic review experts. The review is registered with the PROSPERO international prospective register of systematic reviews (registration number CRD42012003428).

### Search strategy for the review

1. Participants: Studies of non-institutionalized adults with one or more chronic conditions will be included. Chronic conditions are defined as conditions that last, or are expected to last, one year or longer and result in functional limitations and/or require ongoing medical care [31]. A published list of common chronic conditions will be included with the study eligibility forms to guide reviewers [7].

2. Interventions: Original reports of randomized controlled trials published between January 2002 and August 2012 seeking to improve the adoption of and adherence to diet/nutrition modification, physical activity, or both. These modifiable behaviors were selected because they are the leading causes of chronic conditions and death in the USA [32]. To focus on the role of healthcare practitioners in health promotion, we will exclude community and environmental interventions (for example media campaigns, legislative measures) and only include physical or virtual interventions delivered from the healthcare setting (primary care, hospital, specialty care, pharmacy, or public health clinic) by healthcare providers/practices for their patients.

3. Control interventions: Studies with either alternate interventions or control interventions (usual care or no intervention) will be included.

4. Outcomes: Outcomes of interest include measures of adherence to one or more of the selected health behaviors.

An expert reference librarian will design and conduct the initial search in relevant biomedical databases, including Ovid MEDLINE, Ovid EMBASE, Ovid PsycINFO, Ovid Cochrane CENTRAL, CINAHL and Web of Science in collaboration with content matter experts. Search terms will include controlled vocabulary and text-words (including truncations) for the following concepts: chronic disease, comorbidity, multimorbidity, adherence, compliance, health behaviors, physical activity, diet, weight loss, and behavioral and educational interventions. We will review the citation and reference sections of eligible studies and available reviews. We will also identify additional references through consultation with content experts, and hand searching of key journals and meeting proceedings.

### Study eligibility

All abstract and full text eligibility and data extraction procedures will be conducted using DistillerSR systematic review software (Ottawa, ON, Canada). Initially, the potential eligibility of each of the abstracts and titles that result from executing the search strategy will be reviewed in duplicate using a pre-defined abstract eligibility form detailing the selection criteria. Full text versions of all potentially eligible studies will be requested. Any disagreements by reviewers will also be retrieved in full text for evaluation. Full text articles (all available versions of each study) will also be independently reviewed in duplicate for eligibility. The reviewers will calibrate their judgments using a smaller set of reports. Subsequently, disagreements will be resolved by consensus; if not possible, by arbitration. Agreement will be measured using the kappa or phi statistics, as appropriate (the latter is appropriate when the distribution of agreement is extreme).

### Data extraction

Data extraction will include full description of participants enrolled, eligibility criteria, behaviors targeted, interventions received, control or alternate interventions, and measures of behavior. To better understand the range of interventions and their effectiveness, characteristics of the interventions will be detailed, including use of behavior of change theories, modes of delivery, orientation of the intervention (towards patient or provider), length of intervention, and other common intervention techniques. Full descriptions of the interventions and outcomes will be collected for further analysis using the CCM (see Analysis section). To ensure the quality of data extraction, each reviewer will be trained on the extraction process and each will extract data from five studies in duplicate to ensure reviewer agreement. Conflicts will be resolved by consensus and this calibration process will be repeated until reviewers reach near perfect agreement. In addition to behavioral outcomes of interest, other significant results indicating improved patient capacity will also be recorded (for example quality of life, clinical outcomes).

### Methodological quality

To assess the methodological quality of randomized trials we will determine the following: how the randomization sequence was generated; how allocation was concealed, whether there were important imbalances at baseline; which groups were blinded (patients, care givers, data collectors, outcome assessors, data analysts); any monitoring for fidelity to the intervention, the loss to follow-up; whether the analyses were by intention to treat; and how missing outcome data was handled. Assessors of quality will work independently and their interobserver agreement optimized through training.

### Data extraction

In order to apply the CCM to this analysis, intervention components will be assigned to the model in a two-step process. Initially, the pre-determined intervention components included in the data extraction form will be fit to the CCM *a priori*. Next, full descriptions of the interventions will be extracted and reviewed to determine how additional elements of the interventions fit into the conceptual model. To determine how intervention components fit the CCM, a review group consisting of physician-researchers, social-behavioral scientists and content matter experts will independently code each intervention component and outcome to the CCM. Inter-rater reliability will be assessed and reported. Disagreements will be resolved by group consensus. To avoid bias, the group categorizing intervention components will be blinded to the outcomes of the studies and will only be presented with the extracted relevant data needed for making the decision. Per the CCM, intervention components will be described as contributing to the patients’ treatment workload, bolstering their capacity, or neutral using the following criteria: workload consists of demands; demands, in turn, are actions that take up time, space, and effort. If intervention components add to these, in terms of traveling a distance, using an amount of time and expending effort (at least two of the three for the purposes of this project) beyond no intervention, then they add to workload. If components somehow decrease at least two of these three factors, then the components decrease workload. If they do not change at least two of these three factors in the same direction, then they will be listed as ‘neutral’ on workload. Intervention outcomes will be assessed the same way. Reviewers will also note whether any study outcomes are in fact related to workload.

Capacity consists of physical, psychosocial, interpersonal, financial, and healthcare-related abilities and resources. If intervention components directly increase or decrease any of these (for example providing diet/exercise counseling is adding to healthcare-related resources available to patients), then they will be rated as increasing or decreasing capacity. Study outcomes, at whatever time of follow-up, will be assessed the same way. Reviewers will also note whether any study outcomes are in fact related to capacity.

We aim to categorize interventions as increased, decreased, or neutral for workload and capacity for each intervention component and measured outcome. This conceptual exercise will not only provide insights into the work required of patients in behavior interventions, but also advance our understanding of how the initial work required of health behavior interventions may in turn increase patients’ capacity as evidenced in intermediate outcomes, and whether it thereby results in significant behavior changes or improved clinical outcomes.

### Meta-analysis

For each study, we will estimate the odds ratio (OR) as the effect size establishing the association between the interventions and adherence to the health behaviors of interest. ORs will be pooled across studies using the random effects model [33] as implemented in Stata version 12 software (StataCorp, College Station, TX, USA). Heterogeneity across individual studies will be assessed using the I2 index and Cochran’s Q statistical test [34]. Meta-regression will be used to test for interactions between the effect size and *a priori* determined covariates (subgroup analysis).

These subgroup analyses will evaluate interactions across these outcomes of intervention effects and: a. number of chronic conditions; b. presence of depression (if depression was required for study inclusion); c. the health behavior targeted; d. whether the intervention was provider-facing or patient-facing; e. whether it was a single- or multiple-risk factor behavior intervention; and f. the conceptual model characteristics of the intervention, including capacity-enhancing and workload-inducing components determined by the group consensus. We will test univariate and multivariate models. Data that are heterogeneous or inappropriate for meta-analysis will be evaluated using a meta-narrative approach.

## Discussion

The results of this review will inform researchers and practitioners as to how clinical health promotion interventions impact health behaviors of adults with chronic conditions. Our unique analyses will give additional insight into how interventions contribute to the patients’ workload of healthcare demands and/or bolster patients’ capacity to better manage those demands. We postulate that interventions will be more successful if they consider the existing capacity and workload of patients with chronic conditions and seek to enhance patients’ capacity for performing health behaviors without adding an unsustainable workload of demands.

The experience of adjusting to a chronic condition brings hardships and subsequent adaptations and resilience [35]. Intervention designs that capitalize on, support, and build patients’ capacity to routinize and adapt to their chronic conditions and successfully implement behavior change into their lives may prove more successful. Clearly, initial increases in a patient’s workload may be required for enactment of health behavior change; however, this workload may increase their capacity to manage their conditions, resulting in decreased burden of illness.

### Strengths and limitations

A primary strength of this study is the application of a patient-centered model to the analysis of the reviewed studies, which may give new insights into how patients with chronic conditions respond to health behavior interventions. This unique analysis will help us describe the type of capacity needed for patients to be successful in modifying diet and physical activity behaviors.

Due to the novelty of our analysis, this review will also face several limitations. Interventions and results may not be reported adequately or in detail, limiting our ability to apply the CCM and make conclusions about its utility. Patient-level data, including personal capacity and social capital [36], will often not be measured and reported. Furthermore, while we intend to compare whether interventions differentially affect patients with single disease versus multimorbidity, we acknowledge that multimorbidity will often be unreported in studies of patients with single chronic conditions, thus it may be difficult to make this comparison. Although we will be limited by our lack of patient-level data, we will be able to explore the application of the CCM to behavior interventions, and begin to illuminate the relationship between patient capacity, treatment workload and the uptake of healthy lifestyle behaviors.

Healthy lifestyle behaviors among patients with chronic conditions can improve patient outcomes, lead to clinically meaningful results, and reduce costs and burden on the healthcare system. This analysis of intervention components impacting diet and physical activity adherence across chronic disease types will lead to better understanding and design of common approaches, which healthcare providers can use when addressing the multiple risk factors that contribute to the burden of chronic conditions. Importantly, this innovative analysis of intervention components regards the patient at the center of clinical health promotion efforts.

## Abbreviations

CCM: cumulative complexity model; OR: odds ratio.

## Competing interests

The authors declare no competing interests.

## Authors’ contributions

AB, NS and VM conceptualized the study, designed the study protocol and drafted the manuscript. HM contributed to the design of the study protocol, search strategy and manuscript. FM and CM contributed to the design of the study protocol and manuscript. PE contributed to the study design and executed the search strategy. All authors read and approved the final manuscript.
